# A COMPARISON OF HEARING AID USERS AND NONUSERS AMONG PERSONS LIVING WITH COGNITIVE IMPAIRMENT

**DOI:** 10.1093/geroni/igae098.2993

**Published:** 2024-12-31

**Authors:** Jayeong Kim, Yeji Hwang

**Affiliations:** Seoul National University, Seoul, Republic of Korea; Seoul National University, Seoul, Republic of Korea

## Abstract

Hearing impairment is common in older adults, including those with cognitive impairment. While hearing aids can alleviate the challenges posed by hearing impairment, less is known about the usage of hearing aids among individuals living with cognitive impairment. Therefore, this study was conducted with two main

**objectives:**

To identify the prevalence of hearing aid usage among individuals with both cognitive and hearing impairment, and to examine the characteristics of those utilizing hearing aids. Secondary data analyses were conducted using 2020 National Survey of Older Koreans, which includes a nationally representative sample of community-residing older adults. This study included older adults aged 65 years and older who had both cognitive and hearing impairment (n=755). Chi-square tests and independent t-tests were employed to compare the characteristics of hearing aid users to non-users. Among those experiencing both cognitive and hearing impairment, only 32.8% used hearing aids. Individuals using hearing aids were older (t=-4.69, p<.001) and had a higher number of chronic diseases (t=-2.10, p=.036) and medications (t=-2.57, p=.010) compared to non-users. Furthermore, hearing aid users exhibited poorer cognition (t=4.17, p<.001), reported greater depressive symptoms (t=-3.7247, p<.001), and participated less in leisure activities (Chi2=11.73, p<.001). Given that only a small portion of individuals facing hearing difficulties utilized hearing aids, there is a need for greater access to these devices to enhance overall quality of life. Additionally, this study revealed that individuals using hearing aids had poorer health conditions compared to non-users. Future research should consider objectively measuring hearing impairment to better examine this relationship.

